# DNA Damage Induced in Glioblastoma Cells by I-131: A Comparison between Experimental Data and Monte Carlo Simulation

**Published:** 2012-06-13

**Authors:** Ali Neshasteh-Riz, Fereshteh Koosha, Afshin Mohsenifar, Seyed Rabee Mahdavi

**Affiliations:** 1. Department of Radiology Technology, Faculty of Allied Health,Tehran University of Medical Sciences, Tehran, Iran; 2. Department of Medical Physics, Faculty of Medicine, Tehran University of Medical Sciences, Tehran, Iran; 3. Department of Toxicology, Faculty of Medical Sciences, Tarbiat Modares University, Tehran, Iran

**Keywords:** Glioblastoma, Monte Carlo Method, Iodine-131, DNA Damage

## Abstract

**Objective:**

The passage of ionizing radiation in living cells creates clusters of damaged nucleotides in DNA. In this study, DNA strand breaks induced by the beta particle of iodine-131 (I-131), have been determined experimentally and compared to Monte Carlo simulation results as a theoretical method of determining^131^I damage.

**Materials and Methods:**

In this experimental study, in order to create single strand breaks (SSB) and double strand breaks (DSB) in the DNA, glioblastoma (GBM) cells were exposed to 10 mCi I-131, at a dose of 2 Gy. Damage of irradiated cells were evaluated quantitatively by the Fast Micromethod assay. The energy spectrum of electrons released in cells were obtained by the macroscopic Monte Carlo code (MCNP4c) and used as an input of the micro Monte Carlo code (MCDS). The percent of damage induced in cells was analyzed by Mann-Whitney test.

**Results:**

A significant reduction (p<0.05) in fluorescence intensity in irradiated cells compared to control cells as determined by the Fast Micromethod assay represented induced SSB and DSB damages in the DNA of irradiated cells. Comparison of experimental and theoretical results showed that the difference between the percentages of SSB per Gy was about 7.4% and DSB was about 1% per Gy.

**Conclusion:**

The differences in experimental and theoretical results may be due to the algorithm of applied codes. Since the Fast Micromethod and other experimental techniques do not provide information about the amount of detailed and complex damages of DNA-like base damages, the applied Monte Carlo codes, due to their capability to predict the amount of detailed damages that occur in the DNA of irradiated cells, can be used in *in vitro* experiments and radiation protection areas.

## Introduction

Deposition of energy by ionizing radiation leads to irreparable damages in the cell nucleous. In particular, DNA is the primary site for damage that is caused by the interaction of ionizing radiation (1). Direct or indirect interaction of ionizing radiation induces a variety of molecular damages, such as single strand breaks (SSB),double strand breaks (DSB), base damage (BD) of various types, and DNA-protein cross links (2, 3)

Some of these damages (SSBs) can be repaired by cellular repair mechanisms, however others (DSBs) are difficult to repair, possibly leading to mutation or cell death (4, 5). Cancer cells are more sensitive to ionizing radiation than normal cells, thus the effects of radiation can destroy cancerous cells. In this way, ionizing radiation is a useful therapeutic tool.

Iodine-131 (I-131) is one of the isotopes that has been extensively used as a beta emitter in radiation therapy. I-131 is known to cause mutation and death in cells that it penetrates, and in other cells at distances of up to several millimeters. Since about 10% of its energy and radiation dose is via gamma radiation, I-131 is used in nuclear medicine imaging techniques. It has been successfully used in the treatment of thyroid cancer and it is considered in the treatment of CNS tumors such as glioblastoma (GBM) (6).

In the past two decades, track structure calculations have contributed to the understanding of mechanisms by which radiation affects cellular systems, and have been applied in nuclear medicine, scanning electron microscopy, radiation therapy, and space radiation biology (7, 8).

In this study, we used I-131 to induce SSB and DSB on GBM cells. In order to determine the differences between the experimental data and theoretical results, the Monte Carlo method was used to simulate the biological consequences of I-131 on DNA, such as the amounts of SSB and DSB, by applying the Monte Carlo (MCNP4C) code and the micro Monte Carlo damage simulation (MCDS) code as proposed by Semenenko and Stewart (9, 10).

## Materials and Methods

### Cell line

U87MG, a human GBM cell line from Pasteur Institute of Iran, was cultured in minimal essential medium (MEM; Gibco/Invitrogen,USA) that contained 10% fetal bovine serum (FBS; Gibco/Invitrogen,USA) and 500 U/ml of penicillin (Sigma, Germany).

### Monolayer culture

GBM cells were cultured as monolayers in T-25 flasks (NUNC) at 37℃, 5% CO_2_ and 95% humidity. During subculturing, we used phosphate buffered saline (PBS) for washing cells; 1 mM 0.25% EDTA was used for trypsinizing the cells.

### Cell irradiation by beta particles

For one flask of monolayer cultured cells, a solution of 10 mCi I-131 in NaOH (0.2 M) was injected into the medium. The flask was then slowly shaken to achieve a homogeneous distribution of I-131 solution around the cells. The flask of GBM cells were exposed for 108 minutes to determine the correlation between DNA damages and the absorbed dose of 2 Gy. At the end of the exposure, the medium was removed and cells were washed and centrifuged by PBS for removal of I-131.

### Fast micromethod assay

Radiation induced SSB and DSB damages in the DNA of GBM cells were evaluated by the Fast Micromethod DNA SSB Assay according to the protocol by Schröder et al. (11). The solutions used to denature DNA were as follows:

Fluorescent dye stock solution (solution A) was the PicoGreen dsDNA quantitation reagent (Molecular Probes). Calcium- and magnesium-free PBS (Ca/Mg-free PBS; solution B) consisted of 137 mM NaCl, 2.7 mM KCl, 4.3 mM Na2HPO4, and 1.5 mM KH_2_PO_4_. Lysing solution (solution C) contained 9.0 M urea, 0.1% SDS, 0.2 M EDTA, at pH 10 with NaOH. Lysing solution supplemented with PicoGreen (solution D) consisted of 20 µL of the original stock dye/mL of solution C. EDTA solution (solution E) contained 20 mM EDTA. NaOH stock solution (solution F) consisted of 1.0 M NaOH and 20 mM EDTA. Working NaOH solution (solution G) was prepared fresh prior to use. A total of 2 mL of solution F was added to 18 mL of solution E and the pH was checked and should be 12.40 ± 0.02.

In order to determine the SSB induced in GBM cells, 3 falcon pipes that contained 100 µl control group cells, (non irradiated cells), were prepared with 4 ml of solution D; 3 other falcon pipes that contained 100 µl of irradiated group cells were also prepared with 4 ml of solution D. In order to lyse the cells, these groups were placed in the dark for 40 minutes. After 40 minutes, the fluorescence intensity of each group was measured with a shimadzu spectrofluorometer using a 485 nm excitation wavelength and 600 nm emission wavelength.

Next, 250 µl of solution G was added to the lysed cells in each group (control and irradiated). The amount of SSB were determined after 20 minutes by measuring the fluorescence intensity of each group.

### Preparing the calibration curve

In order to determine the amount of fluorescence intensity for digestion of all the DNA, 50 µl of DNase, 3 ml of solution D, and 1 ml PBS were added to 100 µl of non-irradiated GBM cells. After 40 minutes the fluorescence intensity was measured using an excitation wavelength of 485 nm and emission wavelength of 600 nm.

### Monte Carlo simulation

To estimate the initial spectrum of secondary electrons produced in a biological target, MCNP4C code was applied, and a monolayer cell culture geometry established. To model a layer of cells attached to the bottom of a culture dish, a cylinder with a diameter of 2.82 cm and a height of 0.4 cm was used. Cells and culture medium were approximated by water at a density of 1.0 g cm^-3^. To estimate particle fluence in a layer of cells attached to the bottom of the culture dish, the particle fluence was tailed in two parallel planes separated by 10 µm. To model the beta source, the probability of beta particle incidence with a variety of energies as shown in table 1 was inserted into the MCNP4c program. The average energy of these electrons was calculated by equation (1), and this average energy was applied in the MCDS code to estimate the percent of damage.

## Results

**Fig 1:**
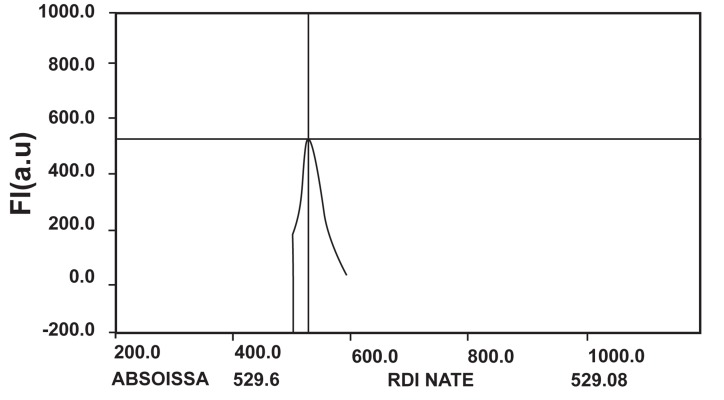
The spectrum obtained from samples of the irradiated and control groups of cells treated with PicoGreen solution, at an excitation wave length of 485 nm and emission wave length of 600 nm (Taken by a Shimadzu spectroflourometer).

The spectrum obtained from samples that were taken by the Shimadzu spectroflourometer are shown in figure1. The average fluorescence intensity obtained from the control and irradiated groups of GBM cells are shown in Table 2. This can be used to determine the amount of SSB and DSB. The average amount of fluorescence intensity in the control and irradiated groups has shown that the absorption of the 2 Gy dose of beta particles of I-131 resulted in fluorescence reduction in the irradiated groups. This reduction represented the existence of SSBs and DSBs in irradiated GBM cells.

**Table 1:** The probability of beta decay in each energy level applied in the MCNP code in order to determine the spectrum of electrons released in medium


Energy (MeV)	0.81	0.47	0.61	0.33	0.25
Probability	0.006	0.005	0.9	0.069	0.02


**Table 2:** The measured average fluorescence intensity of control GBM cells and those irradiated by 10 mCi I-131, to determine the amount of SSBs and DSBs


	DSB	SSB

Average fluorescence intensity (control group)	526.35 ± 22.56	479.4 ± 11.15
Average fluorescence intensity (irradiated group)	482.59 ± 10.20	435.7 ± 18.56


**Fig 2:**
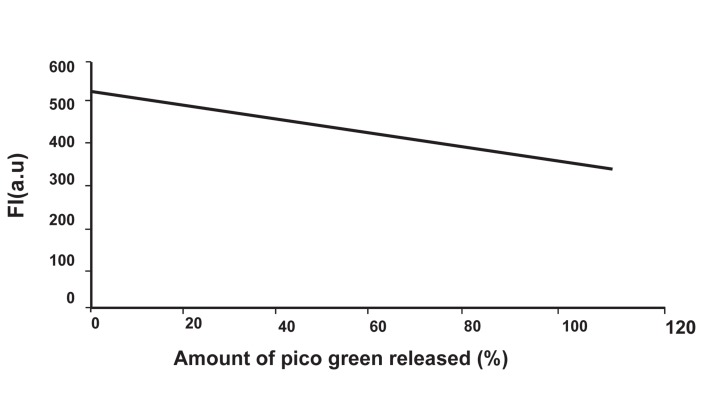
Calibration curve obtained by the amount of fluorescence intensities of control group and DNase-treated group ≈1.79 change in amount of fluorescence intensity, a 1% break will occur.

According to table 2, the average amount of fluorescence intensity in the control group with no DNA damage equaled 526.35±22.56. The average amount of fluorescence intensity in the group treated by DNase equaled 347.4±23.7. According to statistical analysis, the average fluorescence intensity in these two groups (irradiated and control) was significantly different (p<0.05). Reduction of fluorescence intensity represented the maximum break in DNA (100% break). The calibration curve has been drawn and is shown in figure 2.

In order to determine the percent of SSBs and DSBs, we have used the information shown in Figure 2. According to this curve, the variation of intensity per break in the DNA strand equals 178.6, which means, for each 1.786≈1.79 change in amount of fluorescence intensity, a 1% break will occur.

The difference between average intensities in the control and irradiated groups according to table 2 is 43.76 (a.u).

$$\frac{43.76}{1.79}=24.44$$

Thus the amount of DSB is 24.44%.

in the control and irradiated groups according to table 2 is: 43.7 (a.u)

$$\frac{43.7}{1.79}=24.41$$

Thus the amount of single stranded break is 24.41%.

**Fig 3:**
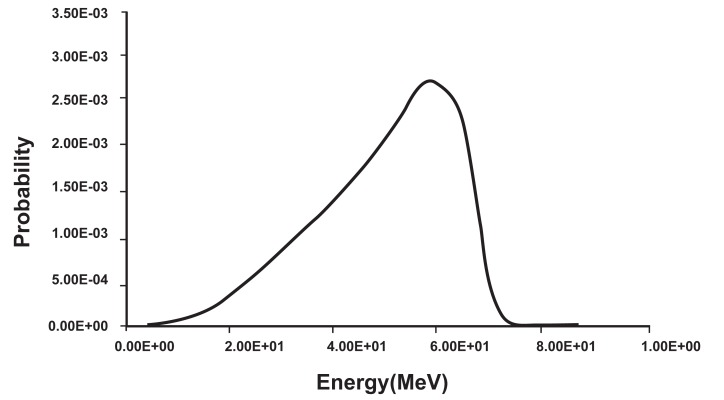
Spectrum of electrons released in the medium obtained by MCNP4c code.

The spectrum of electrons released in the medium obtained by the MCNP4ccode is shown in figure 3, from this spectrum the average energy of electrons calculated by equation 1 is 0.471 MeV.

Equation 1 $$\mathrm{E̅}=\frac{\int_{0}^{E_{\max}}E\phi_{E^{\mathrm{dE}}}}{\int_{0}^{E_{\max}}\phi_{E^{\mathrm{dE}}}}$$

**Table 3:** Percent of different classifications of damage obtained from MCDS output with an electron energy of 0.471 MeV


Clustered damage	Damage yield (%)

BD	68.398
SSB	28.818
SSB +	1.294
2SSB	0.159
DSB	1.154
DSB +	0.158
DSB ++	0.018
SSBc	4.801
SSBcb	40.451
DSBc	13.236
DSBcb	51.237


BD: One or more base damage (no Sb), (SSB), (SSB+) two Sb on the same strand, (2 SSB) two or more Sb on opposite strands separated by at least 10 bp, (DSB), (DSB+) DSB accompanied by one (or more) additional Sb within 10 bp separation, (DSB++) more than one DSB whether within the 10 bp separation or further apart, (SSBc) fraction of complex damage (SSB+ and 2SSB), (SSBcb) fraction of complex damage (SSB+ and 2SSB); base damage included, (DSBc) fraction of complex damage (DSB+ and DSB++), (DSBcb) - fraction of complex damage (DSB+ and DSB++); base damage included (8, 3).

Because both SSBc and DSBc demonstrate more realistic and detailed results of damage in the DNA hit sites, thus they are more comparable to the experimental results. According to the comparison, the difference between the amounts of SSB per Gy is approximately 7.4%, and the difference between the amount of DSB is about 1%.

## Discussion

Low-linear energy transfer (LET) and high-LET radiation create SSB and DSB in a typical mammalian cell (12). In addition, ionizing radiation causes massive amounts of damage to nucleo bases. DSBs are created when at least two strand breaks are formed on opposite strands of the DNA within 10 base pairs. DSBs and other classes of multiple damage sites are the primary cause of radiation-induced cell death (13) and mutagenesis (14). Most experimental techniques used to detect radiation damage to DNA provide only limited information about the exact number and spatial configuration of elementary damages to the DNA. Instead, detailed information about the spectrum of possible damages produced by ionizing radiation is often obtained using Monte Carlo track structure simulation (3, 8, 15). In the past two decades, applying ionizing radiation to induce damages in tumoral cells has been considered a useful tool to cure cancer. I-131 is an important radio isotope of iodine mostly used in the medical and pharmaceutical fields. Moderate doses of I-131 are widely used for curing thyroid cancers. Some special properties of I-131, such as the capability of labeling different kinds of antibodies (MIBG), its short range of beta particles (1 mm) (16), and causing damage through cross-fire phenomena has made it a suitable tool for treating GBM, the most common and most malignant of the glial tumors. Chemotherapy, surgery and radiotherapy are common, but not very successful, ways of treating GBM because CNS tumors are restricted to critical organs. Thus target therapy can be a better choice.

In this study, GBM cells cultured as monolayers were irradiated by I-131, the statistically significant reduction of fluorescence intensity of the cells irradiated by I-131 compared to the control group represented the increase in damages to their DNA. Simulation results by MCNP and MCDS codes has given the percent of different classes of damages induced in DNA by electrons such as DSB, SSB, DSB+, DSB++, 2SSB, etc. In these calculations, it is assumed that a minimum energy of 17.5 eV (17,18) is required for inducing the SSB on a DNA strand, and it is also assumed that a DSB is formed when the breaks on opposite strands are within ≤10 bp separation of each other. To establish criterion for the local energy required to induce SSB, numerous physical and chemical values have been considered such as including average energy loss, ionization energy, oscillator strength, and average excitation potential (ranging from 12eV to 30eV) (18). In table 3 it is noted that inclusion of base damage substantially increases the complex proportion of both SSB and DSB. According to MCDS output in table 3, the amount of DSBcb (complex DSB that includes base damages) is 38% higher than DSBc, and the amount of SSBCb is 35.65% higher than SSBc. Since SSBc and DSBc, demonstrate more realistic and detailed results of damages in hit sites of DNA, they are more comparable to experimental results. According to the comparison, the difference between the amount of SSB per Gy is approximately 7.4% and the difference between the amount of DSB per Gy is about 1%. The difference may be due to the algorithm of the Monte Carlo codes. In order to decrease the discrepancy of the theoretical and experimental results for an individual experiment, a particular code (according to the type of radiation and geometry used) must be designed.

## Conclusion

The determination of induced damages in DNA by the Fast Micromethod assay does not provide information about the percent of complex damages, thus the Monte Carlo codes give more detailed analysis of complex damages induced by radiation in cellular DNA. This information can be used to estimate the amount of damage to irradiated cells in *in vitro* experiments and radiation protection issues.
